# Circulating miR-618 Has Prognostic Significance in Patients with Metastatic Colon Cancer

**DOI:** 10.3390/curroncol28020116

**Published:** 2021-03-15

**Authors:** Maria Radanova, Galya Mihaylova, Zhasmina Mihaylova, Desislava Ivanova, Oskan Tasinov, Neshe Nazifova-Tasinova, Pavel Pavlov, Milko Mirchev, Nikolay Conev, Ivan Donev

**Affiliations:** 1 Department of Biochemistry, Molecular Medicine and Nutrigenomics, Medical University of Varna, 9000 Varna, Bulgaria; galya.mihaylova@mu-varna.bg (G.M.); desiplamenova@gmail.com (D.I.); oskan.tasinov@mu-varna.bg (O.T.); neshe.tasinova@mu-varna.bg (N.N.-T.); 2 Clinic of Medical Oncology, Military Medical Academy, 1000 Sofia, Bulgaria; zhasmina.mihaylova@vma.bg; 3 Department of General and Clinical Pathology, Forensic Medicine and Deontology, Medical University of Varna, 9000 Varna, Bulgaria; pavel.pavlov@mu-varna.bg; 4 Second Department of Internal Diseases, Medical University of Varna, 9000 Varna, Bulgaria; milko.mirchev@mu-varna.bg; 5 Department of Oncology, Medical University of Varna, 9000 Varna, Bulgaria; nikolay.conev@mu-varna.bg; 6 Clinic of Medical Oncology, Hospital Nadezhda, 1000 Sofia, Bulgaria; i.donev@nadezda.bg

**Keywords:** metastatic colon cancer, miR-618, rs2682818

## Abstract

The present study evaluated the prognostic role of circulating miRNA-618 in patients with metastatic colon cancer (mCC) and whether miR-618 gene rs2682818 single nucleotide polymorphisms (SNP) are associated with colon cancer susceptibility and expression levels of mature miR-618. In total, 104 patients with mCC before starting the chemotherapy were investigated. The expression status of circulating miR-618 in mCC was evaluated by quantitative PCR. TaqMan PCR assay was used for rs2682818 SNP genotyping. miR-618 was overexpressed in serum of mCC patients. Patients with high and intermediate expression of miR-618 had a significantly longer mean overall survival (OS) of 21 months than patients with low expression—16 months. In addition, multivariate Cox regression analysis confirmed the association between high/intermediate levels of miRNA-618 and longer OS, HR = 0.51, 95% CI: 0.30–0.86, *p* = 0.012. miR-618 rs2682818 SNP significantly decreased the risk of colon cancer susceptibility in both heterozygous codominant (AC vs. CC, OR = 0.39, 95% CI: 0.17–0.88, *p* = 0.024) and overdominant (AC vs. CC + AA, OR = 0.37, 95% CI: 0.16–0.85, *p* = 0.018) genetic models. Our data suggest that circulating miRNA-618 could be useful as a prognostic biomarker in mCC. Patients harboring AC rs2682818 genotype have a decreased risk for colon cancer in comparison with patients with CC and AA genotypes.

## 1. Introduction

Colorectal cancer (CRC) is the most commonly diagnosed gastrointestinal cancer worldwide, with more than 1.8 million new cases and over 800,000 deaths annually [1]. Despite the fact that 5-year survival rate for patients with CRC has been increased over the past several decades, approximately 25% of patients present with synchronous metastases at the time of diagnosis and almost 50% of patients are expected to develop metastases, resulting in high mortality rates [2]. Therefore, finding novel prognostic markers which can allow detection of patients at risk for poor outcome is crucial.

MicroRNAs (miRNAs) are short (18–25 nucleotides) non-coding RNAs, which can regulate gene expression by suppressing translation or degrade target mRNAs by binding to their 3′-UTR [3]. miRNAs have been suggested as functional biomarkers because of their small size and relative stability. miRNAs are useful biomarkers in early identification of cancer, in monitoring of therapy resistance and in the prognosis of disease outcome [3,4]. Abnormal expression of many miRNAs is well-studied and understood in the context of the occurrence and progression of many different cancers [3,4]. Deregulation of expression of miRNAs may be due to the occurrence of single nucleotide polymorphisms (SNPs) in the genes for miRNAs. A number of studies are concentrated on the clarification of the significance of SNPs connected with cancer risk [5,6]. The released miRNAs in serum are signaling molecules with a definite role in cell–cell communication. Cells from which miRNAs originate can affect the gene expression of adjacent or distant target cells [3].

Several studies show that dysregulation of miR-618 may play a determinant role in the pathogenesis in a number of solid tumors—breast cancer [7,8], hepatocellular carcinoma [9], head and neck squamous cell carcinoma [10], thyroid cancer [11,12,13], prostate cancer [14,15], gastric cancer [16,17], bladder cancer [18], osteosarcoma [19,20] and esophageal adenocarcinoma [21]—and a hematology malignancy—follicular lymphoma [22]. This miRNA has been thoroughly investigated in tumor tissue, and yet, little is known regarding its levels of expression in the blood. Despite the abundance of data about the miR-618 functions in a number of tumors, there are no studies evaluating the expression of miRNA-618 as a diagnostic and prognostic biomarker in CRC.

In this study, we measured the baseline expression of miR-618 in patients with metastatic colon cancer (mCC) before the start of their treatment. We investigated the relationship of miR-618 expression with clinicopathological characteristics and clinical outcome. Moreover, we analyzed whether rs2682818 SNP (C > A) of miR-618 is associated with colon cancer susceptibility and expression level of mature miR-618.

## 2. Materials and Methods

### 2.1. Patients and Healthy Controls

In total, 104 Caucasians patients with unresectable mCC before starting the chemotherapy (CT) were included in the study. The patients were selected and followed-up in Military Medical Academy, Sofia and University Hospital “St. Marina,” Varna. The therapies of patients included first line fluoropyrimidine-based CT (FCT) or FCT in combination with targeted therapy with a monoclonal antibody targeting vascular endothelial growth factor (anti-VEGF) or with a monoclonal antibody against epidermal growth factor receptor (anti-EGFR).

The mean age at the time of diagnosis of the patients was 61.46 ± 10.94. Of the patients, 33 were female (31.73%) and 71 were males (68.27%). In total, 82 (78.85%) of all the patients had a metastasis in liver, 17 (16.35%) in peritoneum and 33 (31.73%) in lungs. Furthermore, for 78 patients (75.00%), the primary tumor was located on the left and 26 (25.00%) on the right colon. Moreover, 48/100 (48.00%) of the patients were positive for *RAS* mutations and 52/100 (52.00%) had wild type *RAS* mutation status. For four patients, there was no information on the presence of *RAS* mutations. There were 10 well-differentiated G1 tumors (9.61%), 76 moderately differentiated G2 tumors (73.08%) and 18 poorly differentiated G3 tumors (17.31%). The patients’ Eastern Cooperative Oncology Group (ECOG) performance status was assessed to be <2. Levels of serum carcinoembryonic antigen (CEA) were assesed in the patients.

As a control group 90 healthy volunteers were included, all Caucasians age and sex-matched to the patients, for the miR-618 expression and SNP genotyping analyses. All healthy controls (HC) had no first-line relatives with a history of colon cancer and no clinical evidence of any other disease.

This was a retrospective study with two approvals of the “Commission for Scientific Research Ethics” of Medical University of Varna, Bulgaria (protocol №74/03.05.2018 and protocol №83/16.05.2019). It was conducted on the leftover serum samples from mCC patients. All patients signed an informed consent for scientific research for the initial bio-bank and data collection according to the Declaration of Helsinki. The authors declared that the research was conducted in accordance with the regulations, which were approved by the ethical standards of the “Commission for Scientific Research Ethics” of the Medical University of Varna, Bulgaria, and with the principles of the World Medical Association Declaration of Helsinki “Ethical Principles for Medical Research Involving Human Subjects” (last revision, October 2013).

Each selected healthy control signed an informed consent form prior to enrollment in the current study. All serum samples from the mCC patients and the HC used in this study were obtained from peripheral blood and stored at −80 °C until use.

### 2.2. RNA Extraction, cDNA Synthesis and qPCR

Small RNA fraction <200 nt, including circulating miRNAs, was extracted from 200 µl serums using NucleoSpin miRNA Plasma kit (Macherey-Nagel GmbH & Co. KG., Düren, Germany). After detection of RNA concentration and purity, 90 ng of RNA was subjected to cDNA synthesis by using Stem-loop primers for miR-618 (5′-CTCAACTGGTGTCGTGGAGTCGGCAATTCAGTTGAGACTCAGAA-3′) and U6 reverse primer (5′-CGCTTCACGAATTTGCGTGTCAT-3′) for the endogenous reference control by RevertAid First Strand cDNA Synthesis kit (ThermoFisher Scientific, Waltham, MA, USA). The quantitative PCR (qPCR) analyses were performed using Luna Universal qPCR Master Mix (New England BioLabs, Ipswich, MA, USA), 3× diluted cDNA template and the following primers: miR-618 forward, 5′-ACACTCCAGCTGGGAAACTCTACTTGTCCTT-3′ and universal reverse, 5′-GTCGGCAATTCAGTTGAG-3′) and U6 (endogenous control) forward, 5′-GCTTCGGCAGCACATATACTAAAAT-3′ and reverse, 5′-CGCTTCACGAATTTGCGTGTCAT-3′. All the qPCRs were performed in triplicate. The relative expression levels were calculated by the 2^−∆∆Ct^ method. The 7500 Real-Time PCR system was used for the qPCR analyses.

### 2.3. DNA Extraction and Single Nucleotide Polymorphism (SNP) Genotyping

Genomic DNA was isolated from plasma using QIAamp DNA Blood MiniKit (Qiagen, Hilden, Germany). SNP rs2682818 miR-618 chr12:80935757 C > A genotyping was performed using a TaqMan allelic discrimination assay (TaqMan Assays SNP Genotyping, ThermoFisher Scientific, Waltham, MA, USA) using the Applied Biosystems 7500 Real-Time PCR System.

### 2.4. Statistical Analysis

Data were managed and analyzed using GraphPad Prism 6.0 software (San Diego, CA, USA). The Mann–Whitney U test, Chi square test and Spearman correlation were used to compare and evaluate the correlations between the levels of miR-618 in serum and clinicopathological characteristics of the patients and the distribution of the genotypes of rs2682818. The Hardy–Weinberg equilibrium was evaluated by Chi-square test, comparing the expected and observed genotype frequencies in control group. Odds ratios (ORs) with 95% confidence intervals (CIs) for categorical outcomes were calculated using the binary logistic regression model. Hazard ratios (HRs) and 95% confidence intervals (CIs) for univariate and multivariate models were calculated using Cox proportional-hazards regression models. Survival curves were estimated using the method of Kaplan-Meier and differences were assessed using the log-rank test. Specificity and sensitivity of serum miRNA-618 expression levels to distinguish mCC patients from healthy controls were evaluated with Receiver Operating Characteristics (ROC) analysis. The diagnostic accuracy of circulating miR-618 was determined by evaluation of the largest possible area under the curve (AUC) in the ROC analysis. Tests with AUC values greater than 0.9 had high accuracy, values between 0.7–0.9 had moderate accuracy and values between 0.5–0.7 had low accuracy [23]. Two-tailed *p*-values (<0.05) were considered as significant.

## 3. Results

### 3.1. Expression of miR-618 in Serum Samples of Metastatic Colon Cancer (mCC) Patients Was Significantly Higher Than in Serum Samples of Healthy Controls

The miR-618 expression was measured by qPCR in the serum of 104 mCC patients and 90 healthy controls. The median expression level of miR-618 in patients was 2.39 (0.008–46.69), the expression in the 33th percentile was 0.75 and the expression in the 66th percentile was 4.66. The median miR-618 expression in healthy controls was 0.15 (0.001–18.36), the expression in the 33th percentile was 0.06 and the expression in the 66th percentile was 0.34.

**Figure 1:**
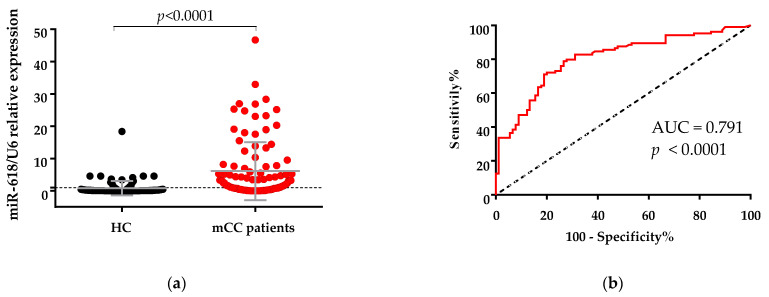
(**a**) Comparison between levels of miR-618 in serum samples from healthy controls (HC) and patients with metastatic colon cancer (mCC) (Mann-Whitney U test, data are presented as mean ± SD). The expression levels were measured using qPCR. U6 RNA was used as an internal control. Relative gene expression was calculated using 2^−∆∆Ct^ method. (**b**) Receiver operation characteristics (ROC) curve of using miR-618 to differentiate patients with mCC from healthy controls. Area under the ROC curve (AUC) is 0.791, *p* < 0.0001.

miR-618 expression in serum was found to be significantly increased in mCC patients’ blood in comparison with healthy controls (*p* < 0.0001, Figure 1a). To evaluate the potential diagnostic value of miR-618, the ROC curve has been used (Figure 1b). At the optimal cutoff value, miR-618 could significantly distinguish between mCC patients and healthy controls with AUC = 0.79 (95% CI: 0.72–0.86, *p* < 0.001) and with a sensitivity of 79.81% and specificity of 72.22%.

### 3.2. miR-618 Serum Levels Was Not Related Significantly with Clinicopathological Characteristics of the Patients

Chi-squared analysis between the levels of circulating miR-618 and clinicopathological characteristics in the patients with mCC was performed (Table 1). Levels of miR-618 were not related to patients’ age at the time of diagnosis (*p* = 0.863), sex (*p* = 0.149), presence of liver (*p* = 0.102), peritoneum (*p* = 0.753) and lung (*p* = 0.422) metastasis, *RAS* mutational status (*p* = 0.721), primary tumor location (*p* = 0.469), grade (*p* = 0.242), PS (ECOG) (*p* = 0.382) and CEA (*p* = 0.437) (Table 1).

### 3.3. Low miR-618 Levels in Serum Were Associated with Short Survival

**Figure 2:**
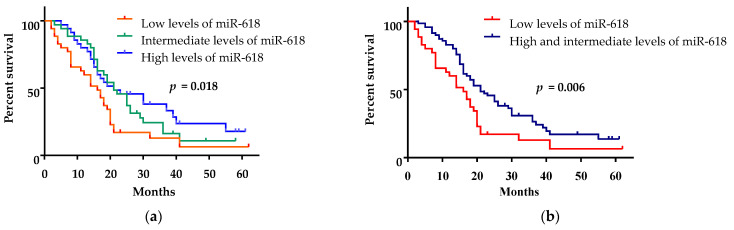
Kaplan–Meier survival analysis for assessment of the circulating miR-618 levels and the overall survival of mCC patients. (**a**) Overall survival of patients with high (over the 66th percentile), intermediate (between the 33th and 66th percentile) and low (up to the 33th percentile) levels of miR-618 were compared. (**b**) Overall survival of patients with high (over the 66th percentile) and intermediate (between the 33th and 66th percentile) level of miR-618 were compared to overall survival of patients with low (up to the 33th percentile) levels of miR-618.

Patients were divided into three groups according to their expression levels of miR-618—with low (up to the 33th percentile, range: 0.008–0.754), intermediate (between the 33th and 66th percentile, range: 0.755–4.661) and high expression (over the 66th percentile, range: 4.662–46.690). The patients with low expression of miR-618 had a mean overall survival of 16 months (95% CI: 11.17–20.83, Figure 2a), while the mean overall survival of patients with intermediate and high expression of miR-618 was the same—21 months (95% CI: 16.44–25.56 and 95% CI: 5.99–36.02, respectively, Figure 2a). Because of their similar mean overall survival, the groups with high and intermediate expression of miR-618 were combined for subsequent analyzes. Patients with low expression of circulating miR-618 had a significantly shorter mean overall survival (16 months; 95% CI: 11.17–20.83) than patients with high/intermediate expression (21 months, (95% CI: 15.83–26.17) (log-rank test, *p* = 0.006, Figure 2b).

In a univariate Cox regression analysis, high/intermediate levels of miR-618 were associated with longer overall survival (HR = 0.56, 95% CI: 0.36–0.89, *p* = 0.013, Table 2). This association was confirmed in the multivariate analysis (HR = 0.51, 95% CI: 0.30–0.86, *p* = 0.012, Table 2).

In the univariate Cox regression analysis, the low grade (HR = 0.58, 95% CI: 0.34–0.99, *p* = 0.048, Table 2) and CEA ≤ 2 ULN (HR = 0.53, 95% CI: 0.33–0.84, *p* = 0.007) were also associated with better outcome, whereas in the multivariate analysis, only CEA ≤ 2 ULN (HR = 0.58, 95% CI: 0.34–0.98, *p* = 0.041, Table 2) remained an independent predictor for longer overall survival.

Patients with low level of expression of miR-618 in serum showed a trend towards shorter mean PFS of 6.15 months (95% CI: 4.41–7.88) as compared to those with high/intermediate expression of miR-618—7.84 months (95%, CI: 6.80–8.86) (log-rank test, *p* = 0.061, data not shown).

### 3.4. The Presence of rs2682818 Did Not Determine Serum Levels of miR-618

**Figure 3:**
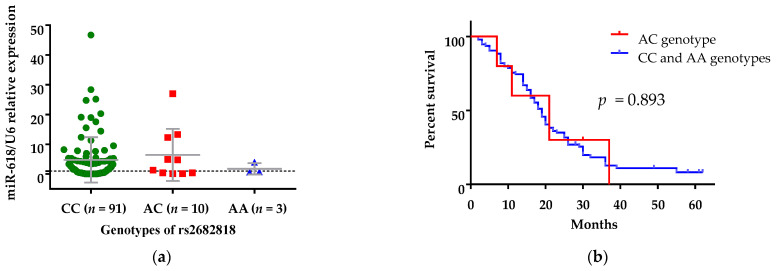
(**a**) Levels of circulating miR-618 in patients with mCC carrying different genotypes of rs2682818. There were no significant differences between rs2682818l genotypes in regard to expression of miR-618 (Mann-Whitney U test, data are presented as mean ± SD). (**b**) Kaplan–Meier survival analysis for assessment of AC vs. CC and AA genetic model of rs2682818 and the overall survival of mCC patients. Patients carrying AA genotype and those carrying CC/AA genotypes did not have statistically different overall survival (*p* = 0.893).

The impact of rs2682818 polymorphism on expression levels of miR-618 was investigated. The levels of miR-618 in patients with different genotypes of rs2682818 polymorphism were compared (Figure 3a). Although levels of circulating miR-618 were numerically lower in patients with the AA genotype, no significant differences were observed between rs2682818 variants with regards to expression of miR-618 (Figure 3b).

### 3.5. rs2682818 AC Genotype Was Associated with a Decreased Risk of Colon Cancer

rs2682818 polymorphism is not investigated for association with CC in Caucasians. Therefore, the distribution of genotypes and alleles frequencies of the rs2682818 were evaluated (Table 3). The frequencies of A and C alleles in patients were similar to frequencies in healthy controls (OR = 0.67, 95% CI: 0.33–1.33, *p* = 0.250, Table 3) and C allele was the most representative in both groups. In patients, the distributions of AA, AC and CC genotype were 0.03, 0.10 and 0.87, respectively (Table 3). We did not find carriers of the AA genotype among the healthy controls, but the distribution of rs2682818 genotypes in this group was in Hardy–Weinberg equilibrium (*p* = 0.590). Statistically significantly decreased risk of CC was found for rs2682818 in heterozygous codominant (OR = 0.39, 95% CI: 0.17–0.88, *p* = 0.024, Table 3) and in overdominant (OR = 0.37, 95% CI: 0.16–0.85, *p* = 0.018, Table 3) genetic models.

Next, a comparison between overall survivals of patients with AC genotype vs. patients with CC/AA genotypes in the overdominant genetic model was conducted. Patients with the AC genotype had no significantly different mean overall survival, i.e., 21 months (95% CI: 9.68–32.32), than patients with CC/AA genotypes—19 months (95% CI: 16.72–21.28) (log-rank test, *p* = 0.893, Figure 3b).

## 4. Discussion

miR-618 deregulation is linked to a number of cancers. There have been several studies that tried to explain the role of miR-618 in cancers, identifying target genes for miR-618 and analyzing their functional relevance. Data have been accumulated about the mechanisms through which miR-618 exerts its effects on underlying molecular pathways in carcinogenesis and cancer progression. In hepatocellular carcinoma [9] and in head and neck squamous cell carcinoma [10], miR-618 inhibits tumor suppressor activity in the cells, while in breast cancer [7], anaplastic thyroid cancer [11,12], gastric cancer [16,17], prostate cancer [15] and osteosarcoma [20], miR-618 inhibits growth, invasion and migration of tumors. Moreover, exogenous miR-618 expression retarded osteosarcoma growth in vivo [20]. These tumor-suppressive or oncogenic functions miR-618 depend on its targeted genes in different cancers. miR-618 has a potential to exhibit dual behavior depending on the context of the disease—context-dependent miRNA. For example, overexpression of miR-618 in vitro in high aggressive prostate cancer cell line increases the capacity for cell invasion and act as oncogene [14]. However, in surgical samples from patients with clinically localized prostate cancer, increased expression suggests a protective function against a more aggressive form of the disease. In thyroid [12], gastric [16,17] and prostate cancer [15], overexpression of miR-618 reduces the PI3K/Akt signaling pathway, inhibits TGF-β or negatively regulates the transcriptional level of TGF-β2. PI3K/Akt pathway is responsible for cellular activities such as cell growth, proliferation, differentiation and migration, and it is a major survival pathway activated in cancers including CRC [24]. Zhu et al. found that metastatic colorectal tumors have a higher frequency of PI3K expression than primary lesions [25]. TGF-β inhibits intestinal epithelium cell proliferation and acts as a tumor suppressor in the early stages of cancer progression [26]. During tumor progression, TGF-β could switch its function from tumor-suppressive to tumor-promoting [27]. The elevated TGF-β levels in primary tumor and in plasma of CRC patients are associated with a higher incidence of distant metastasis and decreased survival. In addition, if miR-618 exhibits similar inhibition effects on the PI3K/Akt pathway or/and on TGF-β in the patients with mCC, it could explain that miR-618 acts as tumor-suppressor miRNA in patients and its low levels imply a progression of the disease.

In the present study, we found that the expression of miR-618 in serum samples of mCC patients was significantly higher than that in serum samples of healthy controls and that circulating miR-618 has a potential to distinguish patients with mCC from healthy controls with moderate accuracy. We also found that low miR-618 levels in serum were associated with short survival in mCC patients.

The studies for the role of miR-618 in different carcinoma were made mainly in cancer cell lines or tumor tissue where its expression level is low. miR-618 is decreased in tumor tissues of patients with breast cancer and prostate cancer and osteosarcoma compared to the adjacent non-tumorous tissue samples; additionally, patients who show low levels of miR-618 in tumor tissue have poor outcomes [7,14,15,20]. According to our knowledge, there have been no investigations on the expression of circulating miR-618 in plasma or serum in patients with solid tumors. In our research, we found that the expression of circulating miR-618 in mCC patients was higher in comparison with the expression in healthy controls, and high and intermediate expression of miR-618 in the serum of patients was associated with a better outcome than in patients with low levels of miR-618.

In an attempt to explain this seeming contradiction, we tested the expression level of miR-618 in a small number of available colon tissue samples from 10 healthy volunteers and tumor tissue samples from 10 mCC patients. We found a trend towards the lower expression of miR-618 in tumor colon tissues in comparison to colon tissues from healthy people (*p* = 0.067, data not shown). Our results were compared with data in two databases—miR-TV (Available online: http://mirtv.ibms.sinica.edu.tw/ (accessed on 5 November 2020)) and OncoMir Cancer Database (Available online: https://www.oncomir.umn.edu/omcd/basic_search.php (accessed on 5 November 2020)), where it was revealed that the expression of miR-618 in the colon adenocarcinoma tissue was significantly decreased in comparison to the surrounding healthy tissue.

High expression levels of miRNAs in circulation and low expression levels in tumor tissue, as in our case, are not unusual results. Recent studies reported that miRNA levels in plasma and tumor tissue showed significant differences. A strong correlation between expression levels of miRNA in colon tumor tissue and levels of fecal miRNAs were found, but not between levels of miRNAs in colon tumor and plasma [28,29].

Higher expression of miR-618 as tumor-suppressor in circulation in comparison with its expression in tumor tissues is not an exception. For miR-148a Tsai et al. found that its overexpression inhibited colon cancer cell proliferation and migration. Moreover, lower levels of miRNA-148a expression in advanced CRC tissues were associated with significantly poorer overall survival rates [30,31]. Both Takahashi et al. and Tsai et al. reported that miR-148a expression is higher in early stage II CRC tissues than in advanced stages and are commensurate with levels of miR-148a in healthy controls. At the same time, miR-148 is strongly upregulated in the serum of CRC patients in a study of Gmerek et al. Unfortunately, the authors did not provide data on whether this overexpression is linked with better survival in patients [32]. In our study, in addition to high serum expression, we indicated the good prognostic significance of miRNA-618 in mCC patients.

Upregulated circulating miR-618 was established in patients with Kawasaki disease [33] during the acute phase of the disease, in serum of patients with type 1 diabetes with microvascular complication [34] and in patients with cardiac sarcoidosis [35].

All these data give us an opportunity to speculate that: 1. If miR-618 is a tumor suppressor, its expression is inhibited in tumor tissue, but its expression in blood is increased compensatory from another source outside the tumor in an attempt to suppress the tumor progression; 2. Probably, miR-618 is not only cancer-related miRNA, but its overexpression in the blood is a result of the internal stimuli in acute inflammatory or advanced disease. Therefore, the generally accepted conception that tumor cells are the main source of circulating miRNAs in patients with cancer may not be quite relevant as an explanation of the high levels in serum of miR-618 and other miRNAs, especially those with tumor-suppression function. Further analyses are needed to prove our assumptions.

The deregulation in expression levels of miR-618 may also be explanted with the appearance of genetic variants in DNA encoding this miRNA. rs2682818 is located within the stem-loop sequence of the miR-618 precursor and probably affect secondary structure and expression of mature miR-618 [36]. To explore whether different levels of miR-618 result from the presence of rs2682818 polymorphism, we performed SNP genotyping of mCC patients and healthy controls. In vitro analysis of rs2682818 show that the A allele leads to decreased levels of mature miR-618 [20]. No significant functional consequences of rs2682818 variants on expression levels of miR-618 were found in our cohort.

Chen [37] found for a Han Chinese population that AA and AC/AA rs2682818 genotypes are associated with a reduced risk of CRC in comparison to the CC genotype. There are no studies among Caucasians populations on the association between the presence of rs2682818 and CRC susceptibility. This study is the first on Caucasians, and in our cohort, individuals with the AC genotype have decreased risk of mCC than individuals with CC/AA genotypes.

We assume that the lower level of miR-618 in mCC patients was probably not determined so much by the presence of rs2682818 SNP, but rather by other inhibitory mechanisms, such as long non-coding RNA, which sponge miR-618 because of dysregulation during the disease. There are studies that examine similar inhibitory interactions between circ-RNAs and miR-618 in gastric and in breast cancers [7,38]. Moreover, Hsiao [39] showed that oncogenic hsa_circ_0001313 (circCCDC66) is overexpressed in colon cancer and promotes tumor growth and metastasis. More research is needed to confirm our findings and assumptions and to explore the potential mechanisms of action of miR-618 in mCC.

In this study, we recognized the significance of miR-618; however, there are still several limitations in our study. First, our study is retrospective and cross-sectional. Second, it is descriptive with an absence of in vitro analyses of the effect of miR-618 on tumor progression. Therefore, more research is needed to confirm our findings and assumptions and to explore the potential mechanisms of action of miR-618 in mCC. Moreover, the expression levels of miR-618 should be investigated in a larger cohort of tissue samples as well as in stage II and III CC patients.

## 5. Conclusions

In this study, we identified for the first time circulating miR-618 in patients with mCC and tried to explain the different expression of miR-618 with the presence of SNP in DNA sequence for miR-618. Our results suggest that the investigation of expression levels of miRNA-618 in serum could be useful as a non-invasive prognostic biomarker in mCC patients. Furthermore, finding that patients harboring the AC rs2682818 genotype had a decreased risk for CC could be used in molecular screening programs for the evaluation of patients at risk of developing the disease.

## Figures and Tables

**Table 1:** Relations between miR-618 expression and clinicopathological characteristics of the mCC patients.

Clinicopathological Characteristics	High/Intermediate Expression of miR-618 Number (%)	Low Expression of miR-618 Number (%)	*p*-Value
Age			0.863
<65	42 (66.7)	21 (33.3)	
≥65	28 (68.3)	13 (31.7)	
Sex			0.149
female	19 (57.6)	14 (42.4)	
male	51 (71.8)	20 (28.2)	
Liver Metastasis			0.102
yes	52 (63.4)	30 (36.6)	
no	18 (81.8)	4 (18.2)	
Peritoneum Metastasis			0.753
yes	12 (70.6)	5 (29.4)	
no	58 (66.7)	29 (33.8)	
Lung Metastasis			0.422
yes	24 (72.7)	9 (27.3)	
no	46 (64.8)	25 (35.2)	
*RAS*			0.721
WT	34 (65.4)	18 (34.6)	
M+	33 (68.8)	15 (31.3)	
Primary Tumor Location			0.469
left colon	54 (69.2)	24 (30.8)	
right colon	16 (61.5)	10 (38.5)	
Grade			0.242
low	60 (69.8)	26 (30.2)	
high	10 (55.6)	8 (44.4)	
PS (ECOG)			0.382
0	31 (72.1)	12 (27.9)	
1	39 (63.9)	22 (36.1)	
CEA			0.437
≤2 ULN	26 (72.2)	10 (27.8)	
>2 ULN	44 (64.7)	24 (35.3)	

M+—positive for RAS mutations; WT—wild type; PS (ECOG)—performance status (Eastern Cooperative Oncology Group); ULN—upper limit of normal; CEA—Carcinoembryonic antigen.

**Table 2:** Results of the Cox regression analysis for predicting the overall survival.

Variable	Univariate Analysis			Multivariate Analysis		
	Hazard Ratio	95% CI	*p*-Value	Hazard Ratio	95% CI	*p*-Value
Age						
<65 vs. ≥65	1.25	0.80–1.95	0.322	1.22	0.72–2.08	0.461
Sex						
male vs. female	1.19	0.75–1.89	0.467	0.68	0.38–1.20	0.182
Liver metastasis						
no vs. yes	0.76	0.44–1.31	0.318	0.77	0.41–1.47	0.773
Peritoneum metastasis						
no vs. yes	0.86	0.49–1.49	0.58	0.69	0.36–1.34	0.27
Lung metastasis						
no vs. yes	1.03	0.66–1.62	0.884	0.93	0.53–1.61	0.783
*RAS* status						
M+ vs. WT	1.06	0.68–1.66	0.782	0.99	0.61–1.60	0.969
Primary tumor location						
left colon vs. right colon	0.93	0.56–1.53	0.768	0.95	0.53–1.71	0.874
Grade						
low vs. high	0.58	0.34–0.99	0.048	0.6	0.33–1.11	0.104
PS (ECOG)						
0 vs. 1	0.64	0.41–1.007	0.053	0.65	0.38–1.10	0.107
CEA						
≤2 ULN vs. >2 ULN	0.53	0.33–0.84	0.007	0.58	0.34–0.98	0.041
miR-618						
high/intermediate vs. low expression	0.56	0.36–0.89	0.013	0.51	0.30–0.86	0.012

M+—positive for RAS mutations; WT—wild type; PS (ECOG)—performance status (Eastern Cooperative Oncology Group); ULN—upper limit of normal; CEA—Carcinoembryonic antigen.

**Table 3:** Genotypes distribution and allele frequencies of rs2682818 in mCC patients (*n* = 104) and healthy controls (*n* = 90). Association of rs2682818 SNP with colon cancer.

rs2682818	mCC Patients, Number	Healthy Controls, Number	Odd	95% CI	*p*-Value
	(Proportion)	(Proportion)	Ratio		
Alleles			0.67	0.33–1.33	0.25
C	192 (0.92)	160 (0.89)			
A	16 (0.08)	20 (0.11)			
Genotypes			0.39	0.17–0.88	0.024
Codominant model					
CC	91 (0.87)	70 (0.78)			
AC	10 (0.10)	20 (0.22)			
AA	3 (0.03)	0 (0.00)			
Dominant model			0.5	0.23–1.07	0.076
CC vs.	91 (0.87)	70 (0.78)			
AC and AA	13 (0.13)	20 (0.22)			
Overdominant model			0.37	0.16–0.85	0.018
CC and AA vs.	94 (0.90)	70 (0.78)			
AC	10 (0.10)	20 (0.22)			

Allele and genotype frequencies are shown in parentheses. OR—odd ratio; CI—confidence interval.

## Data Availability

The data presented in this study are available on request from the corresponding author at e-mail: maria.radanova@mu-varna.bg. The data are not publicly available due to their containing information that could compromise the privacy of research participants.
